# Fracture Resistance of Anterior Crowns Reinforced by Short-Fiber Composite

**DOI:** 10.3390/polym14091809

**Published:** 2022-04-28

**Authors:** Lippo Lassila, Anssi Haapsaari, Pekka K. Vallittu, Sufyan Garoushi

**Affiliations:** 1 Turku Clinical Biomaterial Center—TCBC, Department of Biomaterials Science, Institute of Dentistry, University of Turku, 20500 Turku, Finland; liplas@utu.fi (L.L.); anssi.haapsaari@utu.fi (A.H.); pekval@utu.fi (P.K.V.); 2 City of Turku Welfare Division, Oral Health Care, 20500 Turku, Finland

**Keywords:** short-fiber composite, load-bearing capacity, indirect conventional composite

## Abstract

The aim of this study was to investigate the load-bearing capacity of anterior crowns made of different commercial particulate-filled composites (PFCs) and reinforced by a core of short-fiber composite (SFC) (bilayer structure). Four groups of composite crowns were fabricated for an upper central incisor (*n* = 20/group). Two groups were made of chair-side PFC composites (G-aenial anterior, GC, Japan and Denfil, Vericom, Korea) with or without SFC-core (everX Flow, GC). One group was made of laboratory PFC composite (Gradia Plus, GC) with or without SFC-core. The last group was made of plain SFC composite polymerized with a hand-light curing unit only or further polymerized in a light-curing oven. Using a universal-testing device, crown restorations were statically loaded until they fractured, and failure modes were visually investigated. Analysis of variance (*p* = 0.05) was used to evaluate the data, followed by Tukey’s post hoc test. Bilayer structure crowns with SFC-core and surface PFC gave superior load-bearing capacity values compared to those made of monolayer PFC composites; however, significant differences (*p* < 0.05) were found in the chair-side composite groups. Additional polymerization has no impact on the load-bearing capacity values of SFC crowns. Using SFC as a core material with PFC veneering composite to strengthen anterior crown restorations proved to be a promising strategy for further testing.

## 1. Introduction

Endodontically treated teeth (ETT) often require substantial build-up with varying post-core foundation materials to retain a complete crown restoration [1]. In recent years, various types of fiber-reinforced composite posts have been introduced in order to provide the dental profession with an alternative to cast or prefabricated metal posts for restoring ETT, as the elastic moduli of these fiber posts are closer to that of dentine than that of metal posts [1]. Many studies have shown that indirect complete crown or overlay restorations and direct composite restorations can be used for final restorations [1,2,3]; however, the best way to restore ETT has long been and still is a subject of debate.

Direct and indirect particulate-filled composites (PFCs) have been successfully used in the clinic for large anterior and posterior restorations [4,5]. Nevertheless, mechanical properties, particularly toughness and polymerization shrinkage, are still issues with contemporary PFCs [6]. Consequently, the clinical performance of PFC restorations is clearly associated with the restoration’s size. Large PFC restorations were more likely to fail due to fractures, resulting in a shorter lifespan [7,8]. The reinforcing phase of restorative PFC has been thoroughly studied with the purpose of improving its toughness and viability for application in high-stress situations. Among the strategies investigated, reinforcing the PFC composite with short (i.e., discontinuous) glass fibers has proven to be one of the most successful [9,10]. Short fibers improved the material’s facility to withstand crack propagation and reduced the stress intensity at the crack tip, where a crack spreads in an unstable way. As a result, an enhancement in composite toughness is observed [11,12]. As a consequence, flowable short fiber-reinforced composite (SFC) was produced a few years ago to address some of the shortcomings and limits of using PFCs in large restorations [13,14]. In comparison to PFC, this SFC was found to have enhanced mechanical properties in terms of fracture toughness and fatigue resistance [13,14].

Many in vitro studies with different applications have looked at bilayer structure composite using SFC as the thick core layer and PFC as the top surface layer [15,16,17]. In these investigations, SFC was used to reinforce extensive posterior composite restorations as core foundations. According to these investigations, the SFC core supports the PFC layer and acts as a crack propagation prevention layer; however, the challenge shows up whether beneficial or not to use the flowable SFC as a core or substructure layer under PFCs (laboratory and chair-side) to construct anterior crown restorations. To the best of the authors’ knowledge, this has not been extensively researched in the literature. Accordingly, this research aimed to study the impact of the SFC core on the load-bearing capacity of anterior composite crown restorations made of different commercial PFCs. The null hypotheses were that (1) the crown restorations with the investigated techniques would have a similar load-bearing capacity and that (2) the type of failure would be unaffected by the restorative technique used.

## 2. Materials and Methods

Table 1 lists the composites utilized in this study.

### 2.1. Crown Fabrication

An experimental abutment (model) for a crown restoration of the upper first incisor was fabricated from Cobalt Chromium blank (Sintermetall, Zirkonzahn GmbH, Gais BZ, Gais, Italy) using a CAD/CAM device (5-TEC, Zirkonzahn GmbH). The abutment was next sintered according to the product instructions in a furnace (Zirkonofen 700/UV, Zirkonzahn GmbH). Except for 2 mm of the cervical area, the metal abutment was then implanted in a plastic tube with self-cured acrylic resin (Palapress, Heraus Kulzer, Wehrheim, Germany). Another dental CAD/CAM device was used to take a photo-impression of the abutment (CEREC, Sirona Dental Systems Inc., Long Island City, NY, USA). An incisor crown restoration was created with a flat surface at the incisor edge in order to adapt to the loading cell during the fracture test.

**Figure 1:**
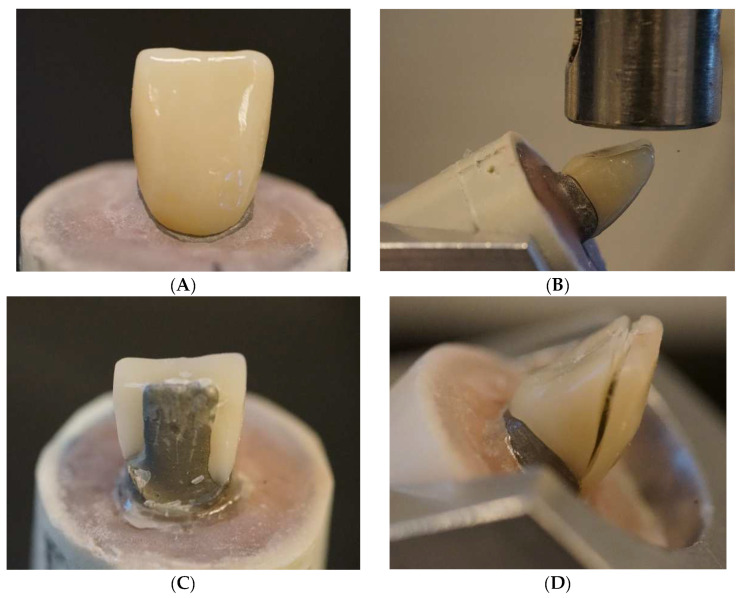
Photographs (**A**–**D**) of test set-up and fracture of a test crown.

A total of 80 manually made anterior composite crown restorations (Figure 1A) were made by one operator and assigned to four groups (*n* = 20/group) according to the technique and material used (Table 2). Two groups were made of chair-side light-cured PFC composites (G-aenial anterior and Denfil) with or without SFC-core (everX Flow). One group was made of laboratory PFC composite (Gradia Plus) with or without SFC-core. The last group was made of plain SFC polymerized with only a hand-light curing unit (Elipar TM S10, 3M ESPE, Seefeld, Germany) for 40 s per segment or further polymerized in a light-curing oven (Targis Power, Ivoclar Vivadent AG, Schaan, Liechtenstein) for 15 min.

For these groups, a transparent template matrix of an appropriately shaped crown (Memosil 2, Heraeus Kulzer GmbH, Hanau, Germany) was employed to help standardized crown restorations. The composite pastes were packed into the space created between the index and the abutment (with or without SFC-core), then light curing was performed using a hand-light curing unit (Elipar TM S10, Seefeld, Germany) for 40 s per segment (wavelength of the light was between 430 and 480 nm and light intensity was 1600 mW/cm^2^). The light source was put in close proximity to the composite surface (1–2 mm). According to the manufacturers’ instructions, Gradia Plus restorations were additionally polymerized in a light-curing oven (Targis Power) for 15 min. The crowns were firmly fixed on the sandblasted and silanated (G-Multi Primer, GC Corp, Tokyo, Japan) metal abutment by only resin without any cement. All crown restorations were polished and stored dry for 48 h at 37 °C before testing.

### 2.2. Fracture Load Test

A universal testing machine (Lloyd model LRX, Lloyd Instruments Ltd., Fareham, UK) was used to apply a quasistatic load to the crown restorations at a speed of 1 mm/min. The acrylic block having the metal abutment and crown was firmly placed on the inclined metal base to offer a 45-degree angle between the loading tip (flat) and the palatal surface of the incisal edge (Figure 1B). Sheets of aluminum foils were positioned above the flat incisor surface in order to load onto the flat incisor surface under the same conditions for all tested crown restorations. The loading event was recorded until restoration fracture (the final decrease in the load–deflection curve), and two investigators visually examined the fracture mode for each specimen.

### 2.3. Microscopic Analysis

Scanning electron microscopy was used to qualitatively investigate representative fractured restorations. (SEM, LEO, Oberkochen, Germany). All of the specimens were cleaned and then coated with a gold layer using a sputter coater in a vacuum evaporator (BAL-TEC SCD 050 Sputter Coater, Balzers, Liechtenstein) prior to observation. The analysis was initiated from the edge of the fractured crown specimen, from the upper PFC loading part to the inner surface and ending at the SFC-core.

### 2.4. Statistical Analysis

To assess the differences between the groups, the data were statistically analyzed with SPSS version 23 (SPSS, IBM Corp., Armonk, New York, NY, USA) using analysis of variance (ANOVA) at the *p* < 0.05 significant level, followed by a Tukey HSD post hoc test.

## 3. Results

**Figure 2:**
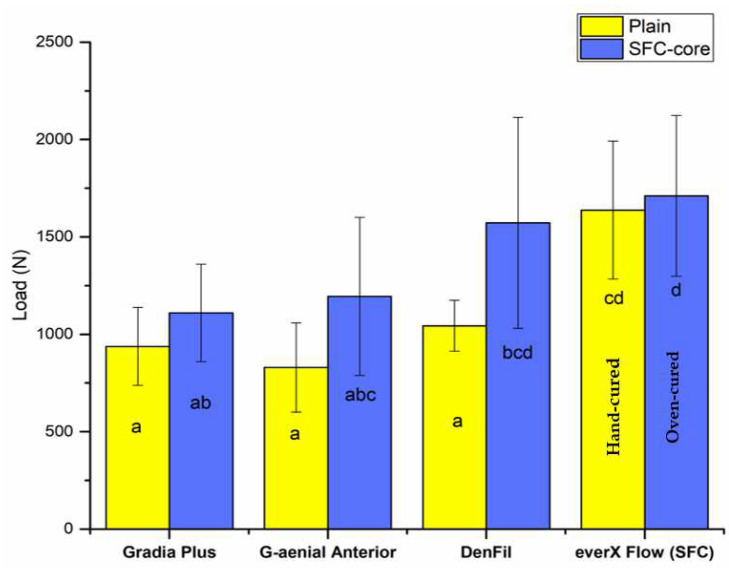
The mean load-bearing capacity values (N) with standard deviation of the tested crowns (plain = monolayer structure; SFC-core = bilayer structure). Non-statistically significant differences (*p* > 0.05) between the materials are represented by the same letters inside the bars.

According to ANOVA, both material type and fabrication technique had a significant (*p* < 0.05) impact on the load-bearing capability of tested crowns, Figure 2 shows the mean (standard deviation) fracture load values of the crowns. According to the findings, restorations reinforced with SFC-core (bilayer structure) exhibited significantly higher load-bearing capacities than monolayer structured composite restorations; however, the significant differences (*p* < 0.05) were found in direct composite groups. Additional polymerization has no impact on the load-bearing capacity values of plain SFC crowns (*p* > 0.05). For all crown restorations (monolayer or bilayer structured), a splitting fracture mode was found during the visual assessment (Figure 1C,D).

**Figure 3:**
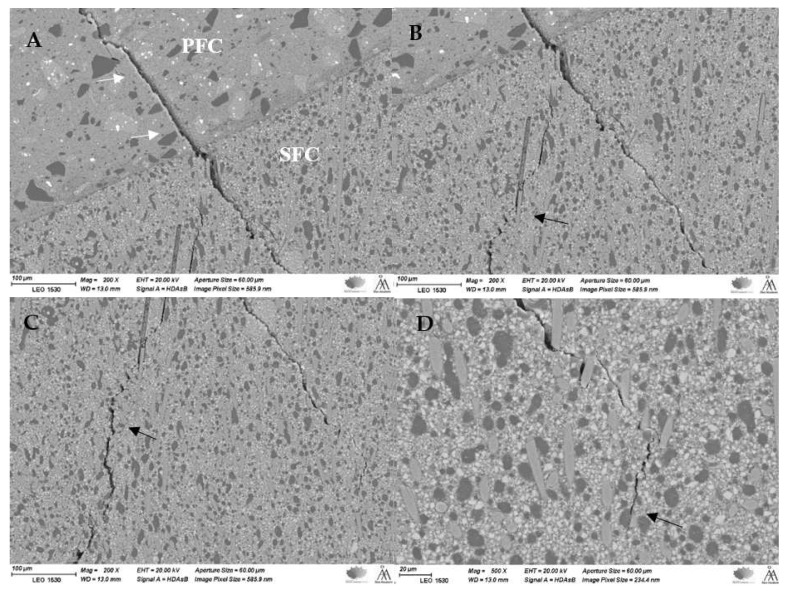
SEM images with different magnifications illustrating a fracture propagation ((**A**), white arrows) from the surface of the PFC composite to the inner part of the SFC-core composite where fibers redirect (**B**,**C**) and stop (**D**) the crack propagation (black arrows).

SEM pictures (different magnifications) of fractured bilayer structured crowns revealed that the fracture crack path propagated radially from the loading surface (incisal) through PFC composite to the inner part at the SFC-core (Figure 3A). Figure 3B–D show how the crack line propagated and then was deflected by fibers, which finally resisted further crack propagation.

## 4. Discussion

The current study compared the effects of two restorative techniques (monolayer structure or bilayer structure with SFC-core) for fabricating anterior crown restorations utilizing different direct/indirect composites.

High fracture toughness and flexural strength have been reported for the flowable SFC (everX Flow) used in this study [13,14]. To our knowledge, there are no other chair-side or laboratory dental composites that have fracture toughness values greater than 2.6 MPam^1/2^. Hence, we hypothesized that a bilayer structure system would improve the load-bearing capacity of anterior composite crown restorations. Our results revealed enhancements in the load-bearing capacity of the restorations when SFC-core was applied in comparison with that of plain (monolayer structure) PFCs (Figure 2). According to the literature, the likely interpretation could be owing to two variables. First, applied stresses could pass from the cross-linked polymer matrix to the glass fibers [9,10], which results in superior load-bearing capacity values. Second, the involvement of individual glass fibers in enhancing the fracture behavior of materials by deflecting cracks [12] (Figure 3), hence increasing the energy required for crack propagation through the polymer matrix or fibers [18,19,20,21].

A number of manufacturers have developed SFCs that claim to overcome the weakness of conventional PFC; however, comparative studies from the literature showed that commercial SFCs have different properties, structures, and reinforcing capacities [22,23]. Based on this, everX Flow was selected for this study.

To gain support from the SFC-core for the PFC, the SFC-core’s toughness must be greater than the PFC surface layer’s [24,25]. In light of this, fiber orientation and properties of the polymer matrix are likely to play a significant role. From another perspective, if the function of the SFC-core is to act as a crack-stopper, the distance between the stress initiation point at the PFC surface and the SFC-core is critical [21]. As a result, the thickness of the surface PFC layer could affect the load-bearing capacity and crack propagation. This is in line with previous research that has shown the importance of how thick SFC and PFC layers should be applied [15,24,26]. Nevertheless, one of our study’s limitations was that the thickness ratio between the SFC-core and PFC was not properly standardized, which explains why certain groups had high standard deviations.

Interestingly, the load-bearing capacity of the reinforced laboratory PFC composite restorations (Gradia Plus) was lower than that of the reinforced chair-side composite restorations (Figure 2). The difference in brittleness between the chair-side and laboratory surface composites over the SFC-core could explain this finding in part—the laboratory PFC composite was additionally polymerized in a light-curing oven, while the chair-side PFC was polymerized by hand light-curing without oven-curing. Consequently, the laboratory-made surface veneering composite was more brittle than the chair-side ones [18]. Another possible explanation is that the evaluated PFC composites (Table 1) have a different amount of filler inclusion, resulting in bearing capacity discrepancies across groups. Additional oven-curing of the plain SFC composite did not improve the performance of the restorations as we expected by improving the degree of monomer conversion. This can be explained by the limiting reinforcing effect of SFC in such applications. In fact, for adequate polymerization of photo-polymerized composites, electromagnetic radiation at the correct wavelength of sufficient intensity and sufficient exposure duration is required [27]. Moreover, SFC has a superior ability to conduct and scatter curing light than traditional PFCs, making it appropriate for usage in bulk with layer thicknesses of 4–5 mm [13,28].

Another aspect that may lead to different load-bearing capacity values is the type of cementation and material used [29]; however, for this study, no specific cement was used and the crowns were fixed solely by resin.

Correlation with the clinical fracture loads is complicated, as the die material (Cobalt–Chromium alloy in our test, dentin in vivo) and its modulus of elasticity will significantly influence stresses within the crowns. This could be considered a major limitation of our study. A metal (Cobalt Chromium) model was used because it aids standardization of the crown abutment and the crown shape, allowing the effect of the material type to be the only variable factor.

An oblique load (45° to the long axis of the tooth) was applied to the tested crowns, which seems to be the worst-case scenario in relation to the load-bearing capacity of anterior crowns [30]. To mimic a more clinical environment and to have a perfect view of material and technique behavior under clinical conditions, long-term water storage and thermal aging should also be taken into consideration.

The ultimate quasistatic force to fracture the crown (final fracture) was determined in this study. Stress applied to dental restorations and teeth is low and cyclic, rather than being static or impact in nature; however, static load is a common approach for testing dental materials used for dental restorations to evaluate their mechanical stability for different applications prior to clinical trials [30,31,32]. Such in vitro investigations help to rank the mechanical and physical properties of new materials; however, more laboratory investigations are needed prior clinical feasibility of the bilayer structure composite crowns.

## 5. Conclusions

Using SFC as core material with conventional PFC veneering composite to strengthen anterior crown restoration proved to be a promising strategy for further testing.

## Figures and Tables

**Table 1:** The used composites.

Material (Code)	Manufacturer	Composition
Gradia Plus (laboratory PFC)	GC Corp, Tokyo, Japan	UDMA, dimethacrylate, inorganic fillers (71 wt%), Prepolymerized fillers (6 wt%)
G-aenial Anterior (chair-side PFC)	GC Corp, Tokyo, Japan	UDMA, dimethacrylate co-monomers, Prepolymerized silica, and strontium fluoride-containing fillers 76 wt%
Denfil (chair-side PFC)	Vericom Corp., Gyeonggi, Korea	Bis-GMA, TEGDMA, 80 wt% Barium aluminosilicate, Fumed silica
everX Flow (SFC)	GC Corp, Tokyo, Japan	Bis-EMA, TEGDMA, UDMA, Short glass fiber (200–300 µm & Ø7 μm), Barium glass 70 wt%

Bis-GMA, bisphenol-A-glycidyl dimethacrylate; TEGDMA, triethylene glycol dimethacrylate; UDMA, urethane dimethacrylate; Bis-EMA, Ethoxylated bisphenol-A-dimethacrylate; wt%, weight percentage.

**Table 2:** Different composite crown restorations (*n* = 20/group).

Material/Group	Technique (Structure)	Polymerization
Gradia Plus	Plain (monolayer)	Hand and oven light-cured
	with SFC-core (bilayer)	Hand and oven light-cured
G-aenial Anterior	Plain	Hand light-cured
	with SFC-core	Hand light-cured
DenFil	Plain	Hand light-cured
	with SFC-core	Hand light-cured
everX Flow (SFC)	Plain	Hand light-cured
	Plain	Hand and oven light-cured
